# VE-PTP controls a fluid shear stress set point that governs cell morphological responses through Tie-2

**DOI:** 10.3389/fcell.2025.1603517

**Published:** 2025-07-04

**Authors:** Keisuke Shirakura, Mana Ghanbarpour Houshangi, Kevin G. Peters, Dietmar Vestweber

**Affiliations:** ^1^ Department of Vascular Cell Biology, Max Planck Institute for Molecular Biomedicine, Münster, Germany; ^2^ Aerpio Therapeutics, Inc., Blue Ash, OH, United States

**Keywords:** VE-PTP, Tie-2, shear stress, cell alignment and elongation, vascular remodeling

## Abstract

Blood flow differs between arteries and veins, hence endothelial cells in these vessels are exposed to different magnitudes of shear stress. Deviation from physiological blood flow triggers vascular remodeling, with increased or decreased flow leading to outward or inward remodeling, to adjust lumen diameter and thereby re-establish physiological shear stress. Based on this, it is assumed that endothelial cells in different vessels differ in their sensitivity to different shear stress levels. Expression levels of VEGFR3 were previously demonstrated to determine the threshold or set point for endothelial cell type specific shear stress sensitivity. Here we show, that the receptor type tyrosine phosphatase VE-PTP and the tyrosine kinase receptor Tie-2 represent another, new signaling system, that determines sensitivity and cellular responsiveness to different shear stress magnitudes or flow set points. We found that increased shear stress levels cause increased levels of VE-PTP endocytosis, which trigger, a similarly graded increase of Tie-2 activity, stimulation of FOXO1 nuclear exclusion and activation of autophagy. The VE-PTP/Tie-2 signaling mechanism controls cell alignment and elongation dependent on the magnitude of shear stress. In addition, VE-PTP/Tie-2 controls shear stress-induced cellular morphological changes independent of VEGFR2. Thus, VE-PTP/Tie-2 is a novel signaling mechanism which determines shear stress sensitivity and morphological responses of endothelial cells.

## 1 Introduction

Endothelial cells sense the magnitude of fluid shear stress generated by blood flow, which contributes to maintaining mechano-homeostasis. Vascular endothelial cells are exposed to varying magnitudes of shear stress depending on the different types of vessels (Lipowsky et al., 1978; Cheng et al., 2003; Reneman et al., 2006). Arterial and capillary endothelial cells experience higher shear stress compared to venous endothelial cells. Under physiological conditions, shear stress is maintained in different types of vessels at different optimal levels described as set point (Baeyens and Schwartz, 2016; Roux et al., 2020). Changing flow magnitude and thus shear stress leads to proportional changes of vessel diameter to restore the initial shear stress levels (Kamiya and Togawa, 1980; Kamiya et al., 1984; Langille and O'Donnell, 1986; Langille et al., 1989; Tronc et al., 1996; Tuttle et al., 2001). Therefore, defects in sensing shear stress magnitudes could potentially lead to vascular malformation (Baeyens et al., 2016; Banerjee et al., 2023). These studies highlight the importance of the ability of endothelial cells to sense a deviation of shear stress magnitude from the physiological set point in order to maintain proper blood flow and vascular function.

Several mechanosensors are known on endothelial cells which convert flow sensing into biochemical signals and cell morphological responses (Aitken et al., 2023). Some of them were shown to be responsible for the varying levels of sensitivity of different types of endothelial cells for fluid shear stress of different magnitudes (Baeyens et al., 2015; Franco et al., 2016; Vion et al., 2020; Banerjee et al., 2023). Vascular endothelial growth factor receptors (VEGFR2 or VEGFR3) are key components of such mechanisms and of a well-defined mechanosensory signaling complex, that relies on PECAM-1, VE-cadherin and a VEGFR (Tzima et al., 2005; Coon et al., 2015). In vascular blood endothelial cells, shear stress-initiated signals are mediated through Latrophilin-2 and PlexnD1, which subsequently lead to the activation of VEGFR2 (Tzima et al., 2005; Mehta et al., 2020; Tanaka et al., 2024), and finally to cellular morphological changes and cell alignment. In contrast to blood endothelial cells, lymphatic endothelial cells are exposed to much lower fluid shear stress levels. It was shown that the sensitivity of lymphatic endothelial cells to low fluid shear stress was due to their high levels of VEGFR3 (Baeyens et al., 2015), which is activated in a similar mechanosensory complex as VEGFR2 in blood endothelial cells (Coon et al., 2015). Variation of VEGFR3 expression levels correlated with higher sensitivity for low shear stress levels (Baeyens et al., 2015). Importantly, altering the expression level of VEGFR3 in zebrafish and mice modulated aortic lumen diameter in agreement with flow-dependent remodeling (Baeyens et al., 2015). Thus, the different expression and signaling level of VEGFR3 provided a molecular basis for a mechanism that could explain the variation of fluid shear stress set points in different types of endothelial cells (Baeyens et al., 2015).

The receptor type tyrosine phosphatase VE-PTP is another endothelial membrane protein which controls endothelial cell responses to fluid shear stress. Exposure to flow leads to the redistribution of VE-PTP to the downstream pole of endothelial cells followed by endocytosis (Mantilidewi et al., 2014; Shirakura et al., 2023). This removes VE-PTP from the tyrosine kinase receptor Tie-2 leading to its activation (Shirakura et al., 2023). Redistribution and endocytosis of VE-PTP was only triggered by laminar flow but not by turbulent flow, and therefore was only seen in atheroprotected and not in atheroprone regions of the aorta. As a consequence, Tie-2 activity increased only at sites of high laminar shear stress which limited leaks and atheroma formation at these sites (Shirakura et al., 2023). Besides their relevance for the regulation of vascular leaks and the development of atherosclerosis, these results revealed that VE-PTP plays a role in converting different types of shear stress (laminar versus turbulent) into distinct activation levels of Tie-2.

Recently, we found that Tie-2 is essential for cell morphological responses and cell alignment to the direction of fluid shear stress (Ghanbarpour Houshangi et al., 2025). In line with this, it was reported, that fluid shear stress promotes the phosphorylation of Tie-2 in a magnitude-dependent manner, although a role in cell alignment was not analyzed (Lee and Koh, 2003). In combination, these two studies allow to speculate that Tie-2 could potentially help endothelial cells to distinguish different magnitudes of fluid shear stress.

In this context it is interesting that we found previously that interference with VE-PTP *in vivo* caused enlargement of venules in juvenile mice (Winderlich et al., 2009). In addition, mutations in Tie-2 which constitutively activate the receptor are a cause of venous malformations, which are selectively found in slow-flow vessels (Vikkula et al., 1996; Wouters et al., 2010; Natynki et al., 2015).

Here, we investigated whether the VE-PTP/Tie-2 axis is indeed required for endothelial cells to distinguish between different magnitudes of laminar fluid shear stress, a function which would be needed to determine the shear stress set point in different vessel types. We found that interference with VE-PTP alters the sensitivity of endothelial cells to different magnitudes of shear stress. In line with this, different magnitudes of shear stress led to different levels of VE-PTP internalization and consequently Tie-2 activation which governed the cell morphological response. Interestingly, these VE-PTP/Tie-2 mediated effecs, and cellular responses were independent of VEGFR2. Collectively, our results suggest that VE-PTP-Tie-2 is a novel determinant of endothelial cell sensitivity for different magnitudes of fluid shear stress.

## 2 Materials and methods

### 2.1 Cell culture

Human umbilical vein endothelial cells (HUVEC) were isolated as described (Wilkens et al., 2024) and cultured in EGM-2 medium (Lonza) at 37°C and 5% CO2 and used for experiments between passages 3 and 6 (Ethics Committee of Muenster University Clinic Approval 2009-537-f-S). Each experiment was reproduced with cells from different donors.

### 2.2 Antibodies

The following antibodies were used for immunofluorescence and immunoblotting: Rabbit monoclonal anti-human FoxO1 (clone C29H4 #2880, Cell Signaling, 1:1,000 for immunoblotting and 1:200 for immunofluorescence staining); rabbit monoclonal anti-human LC3B (clone D11 #3868, Cell Signaling, 1:1,000 for immunoblotting); mouse monoclonal anti-human Tie-2 (clone Ab33, #05-584, Sigma, 1 μg/mL for immunoblotting or 5 μg/mL for immunoprecipitation); mouse monoclonal anti-human α-tubulin (clone B-5-1-2 #T6074, Sigma, 0.5 μg/mL for immunoblotting); mouse monoclonal anti-phosphotyrosine (clone 4G10, #05-321, Sigma, 0.5 μg/mL for immunoblotting); mouse monoclonal anti-human VE-cadherin (clone F-8 #sc-9989, Santa Cruz Biotechnology, 1:100 for immunofluorescence staining); rabbit polyclonal anti-human VEGFR2-pY1054/59 (#44-1047G, invitrogen, 1:250 for immunofluorescence staining) rabbit polyclonal anti-human VE-PTP (VE-PTPh1-8, homemade (Li G. et al., 2020), 10 μg/mL for immunofluorescence staining); rabbit polyclonal anti-human VE-PTP (VE-PTP-C, homemade (Nawroth et al., 2002), 1 μg/mL for immunoblotting). Secondary antibodies conjugated to Alexa Fluor 488 (1:1,000) or Alexa Fluor 568 (1:1,000) and Horseradish Peroxidase (1:5,000–10,000) were purchased from Invitrogen and Jackson ImmunoResearch, respectively. Hoechst 33342, trihydrochloride, trihydrate (H3570) was from Invitrogen.

### 2.3 Inhibitors

To inhibit VE-PTP, HUVECs were treated with 5 μM of AKB-9778 for 4 h before starting and during shear stress exposure.

### 2.4 siRNA

VE-PTP expression in HUVEC was silenced with a combination of two siRNA against VE-PTP (Hs_PTPRB_5, 5′-UAACUUGAUAAAGUCGACCGG-3′ and Hs_PTPRB_10 5′-UAUCGUUCCACAUUCCCAGAA-3′, Qiagen); FoxO1 was silenced with siRNA 5′AAGAGCTGCATCCATGGACAA-3′ (#SI04435144, Qiagen); Tie-2 was silenced with siRNA 5′-TCGGTGCTACTTAACAACTTA-3’ (#SI00604919, Qiagen); VEGFR2 was silenced with a combination of four siRNA (SMARTpool) against VEGFR2 (5′-GGGCAUGUACUGACGAUUA-3′, 5′-CUACAUUGUUCUUCCGAUA-3′, 5′-GGAAAUCUCUUGCAAGCUA-3′ and 5′-GCGAUGGCCUCUUCUGUAA-3′ Dharmacon, # L-003148-00-0005). Allstar negative siRNA (Qiagen) was used as a negative control. siRNA was transfected using Lipofectamine RNAiMAX (Invitrogen) according to the manufacturer’s protocol.

### 2.5 Exposure of HUVEC to shear stress

HUVECs were exposed to shear stress as we previously described (Shirakura et al., 2023). HUVECs were plated on fibronectin-coated ibiTreat surface µ-Slide VI 0.4 (80606, ibidi GmbH), µ-Slide I Luer 0.8 (80196, ibidi GmbH), µ-Slide I Luer 0.4 (80176, ibidi GmbH) or µ-Slide I Luer 0.2 (80166, ibidi GmbH) at a density of 1 × 10^5^ cells/cm^2^ and cultured for 48 h in EGM-2 medium (Lonza). The culture medium was replaced either with EBM-2 medium (Lonza) containing 2% FBS and 1% penicillin/streptomycin, or complete M199 buffer (#11150-59, Gibco) containing 20% FBS, 50 μg/mL ECGS (#354006, Corning), 100 μg/mL heparin and 1% penicillin/streptomycin, or M199 containing 2% FBS and 1% penicillin/streptomycin 4 h before applying cells to shear stress. Shear stress was applied for 24 h (10902 Pump System, ibidi GmbH, Germany) at 37°C in the same humidified incubator. The magnitude of shear stress was calculated according to the manufacturer’s protocol.

### 2.6 Cell-based ELISA in HUVECs

HUVEC on μ-slide VI 0.4 were first exposed to shear stress for 30 min, followed by fixation in 4% PFA in PBS for 15 min, blocking in 1% BSA for 1 hour at room temperature and finally by incubation with 0.5 μg/mL of primary antibodies or isotype control IgG at 4°C overnight. Primary antibodies were detected with peroxidase-conjugated secondary antibody and o-phenylenediamine dihydrochloride (OPD) substrate (Thermo Fisher Scientific). The procedures were subjected to HUVECs attached on the μ-slide. Optical density (OD) at 490 nm was measured with a plate reader (Synergy 2, LabTek). Data in each experiment were normalized to OD490 values in empty lanes.

### 2.7 Immunoprecipitation and immunoblotting

HUVECs on μ-slide I Luer 0.4 were harvested in lysis buffer containing 20 mM Tris-HCl, pH 7.4, 150 mM NaCl, 2 mM CaCl_2_, 1.5 mM MgCl_2_, 1 mM Na_3_VO_4_, 1% Triton X-100, 0.04% NaN_3_ and 2 × cOmplete EDTA-free Protease Inhibitor Cocktail (Roche) and lysed for 30 min at 4°C followed by centrifugation for 30 min, 200,00× g at 4°C. Lysate aliquots were either directly analyzed by immunoblotting (see below), or subjected to immunoprecipitations by incubating with Protein G Sepharose 4 Fast Flow (GE Healthcare) and the respective antibody for 2 h at 4°C. Precipitated immunocomplexes were washed five times with the respective lysis buffer and dissolved in sample buffer (200 mM Tris-HCl pH 6.8, 30% glycerol, 6% SDS, 0.1% bromophenol blue, and 150 mM dithiothreitol) for Western blot analysis. For blotting, either precipitated immunocomplexes or HUVEC cell lysates were separated on 8% SDS-gels and transferred to nitrocellulose (Schleicher & Schuell) by wet blotting. Target proteins were detected by mouse monoclonal Ab33 anti-human Tie-2, rabbit polyclonal VE-PTP-c anti-human/mouse VE-PTP, or mouse monoclonal 4G10 anti-phosphorylated tyrosine. Blocking buffer contained 2% BSA and 200 μM Na_3_VO_4_ instead of 3% skimmed milk when phosphorylated proteins were detected. Phosphorylated Tie-2 was standardized to the expression of total precipitated Tie-2.

### 2.8 Immunofluorescence staining

HUVECs on μ-slide VI 0.4 or I Luer 0.2, 0.4 or 0.8 were fixed with 4% PFA in PBS for 15 min, then permeabilized with 0.3% Triton X-100 in PBS for 5 min, followed by blocking with 1% BSA in PBS for an hour at room temperature. After blocking, the cells were incubated overnight with primary antibodies at 4°C. Subsequently, the cells were washed with PBS and incubated with secondary antibodies and Hoechst for an hour at room temperature. Images were obtained using a Zeiss LSM780 or 880 confocal microscopies.

### 2.9 Analysis of endothelial cell morphology

Cell alignment and elongation were determined after cells were stained for VE-cadherin to define the cell borders. Confocal microscopic images (1,024 × 1,024 pixels, Zeiss LSM 780 or 880 inverted confocal microscope with x20 NA 0.80) were imported into ImageJ/Fiji. To define cell shape, the VE-cadherin channel was applied to “Subtract Background, rolling = 20” and the filter “Gaussian Blur at sigma = 3” followed by converting images to binary images with “Auto threshold with Huang method”. To detect orientation and length of major and minor axis in each cell, we fitted ellipses to the cellular outline in the binary image with “Analyze particle”. The angle of long axis against shear stress direction was considered as cell orientation. We analyzed the orientation and cell elongation index of >500 cells in at least 5 images for each flow chamber lane. Average value in each flow chamber lane was calculated and plotted into graph.

### 2.10 Analysis of FoxO1 nuclear levels

Confocal microscopic images were analyzed with ImageJ Fiji. To prepare the nuclear mask, the Hoechst 33342 signal was applied to the filter “Gaussian Blur at sigma = 1.5”, and then the images were converted to binary images with “Auto threshold with Huang method”. Nuclear FoxO1 signals were extracted by applying the mask to the images of FoxO1 using the “image Calculator”.

### 2.11 Statistical analysis

Values are expressed as mean ± standard error of the mean (SEM). Differences between two groups were assessed by two-tailed Welch’s t-test or Mann-Whitney U-test and among more than two groups were assessed by ANOVA followed by Bonferroni correction test, Dunnett’s test, or Tukey test for multiple comparisons (Prism 10). The statistical significance of differences is shown in figure legends. P values < 0.05 were considered statistically significant.

## 3 Results

### 3.1 VE-PTP silencing affects morphological responses of endothelial cells differently at different shear stress levels

**FIGURE 1:**
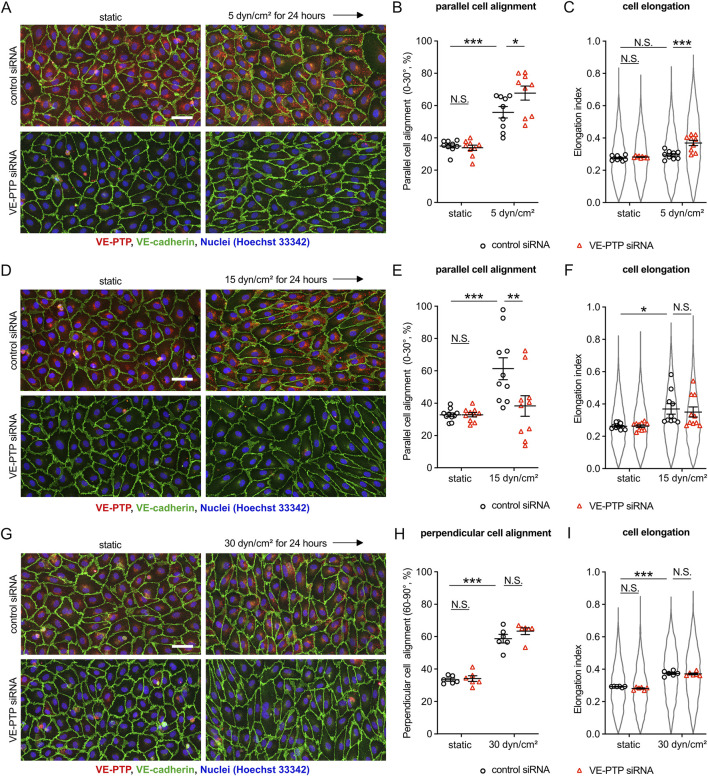
Silencing VE-PTP affects cell alignment and elongation differently at different shear stress levels **(A–I)** Effect of VE-PTP siRNA on cell alignment and cell elongation under 5 dyn/cm^2^ **(A–C)** 15 dyn/cm^2^ **(D–F)** and 30 dyn/cm^2^ **(G–I)** of shear stress. HUVECs transfected with control or VE-PTP siRNA were exposed to indicated shear stress levels for 24 h in EBM-2 flow medium, followed by staining for VE-cadherin (green) and VE-PTP (red) **(A,D,G)**. Percentage of parallel or perpendicular cell alignment **(B,E,H)** and elongation **(C,F,I)** were quantified with Fiji/ImageJ. Mean ± SEM; n = 7 **(A–C)**, 10 **(D–F)** or 6 **(G–I)**; P values are calculated by two-way ANOVA followed by Tukey’s multiple tests **(B,C,E,F,H,I)**. Scale bars: 50 μm **(A,D,G)**.

To investigate the potential role of VE-PTP for cellular morphological responses to fluid shear stress, we analyzed cell alignment and elongation of HUVECs exposed to various magnitudes of shear stress. First, we applied 5 dyn/cm^2^ of shear stress to HUVECs transfected with VE-PTP or control siRNA (Figures 1A–C). In control cells, 5 dyn/cm^2^ of shear stress induced slight cell alignment, but no cell elongation along the direction of flow. VE-PTP silencing, however, promoted cell alignment and cell elongation. The same effects were observed when VE-PTP silencing was replaced by the treatment of cells with the pharmacological VE-PTP inhibitor AKB-9778 (Supplementary Figures S1A–C). These results indicate that VE-PTP suppresses cellular morphological responses to low 5 dyn/cm^2^ shear stress levels, a function which depends on its phosphatase activity.

Next, we applied 15 dyn/cm^2^ of shear stress to HUVECs transfected with VE-PTP or control siRNA (Figures 1D–F). In control cells, 15 dyn/cm^2^ of shear stress induced well detectable cell alignment and elongation along to the direction of flow, whereas VE-PTP silencing suppressed cell alignment, but had no effect on cell elongation. Likewise, AKB-9778 treatment also suppressed cell alignment, but did not affect cell elongation (Supplementary Figures S1D–F). Thus, VE-PTP plays opposite roles for cellular morphological responses to low (5 dyn/cm^2^) and moderate (15 dyn/cm^2^) shear stress levels.

Analyzing high shear stress levels, we exposed HUVEC to 30 dyn/cm^2^ of shear stress. This treatment induced cell alignment and cell elongation perpendicular to the direction of flow which occurred, interestingly, independent of whether VE-PTP was silenced or not (Figures 1G–I). Taken together, VE-PTP silencing increases the sensitivity of endothelial cells to low shear stress levels, reduces the sensitivity to moderate levels and is not relevant for the perpendicular alignment of cells at high levels of shear stress.

### 3.2 The magnitude of shear stress determines VE-PTP cell surface levels, which affects the levels of Tie-2 phosphorylation

**FIGURE 2:**
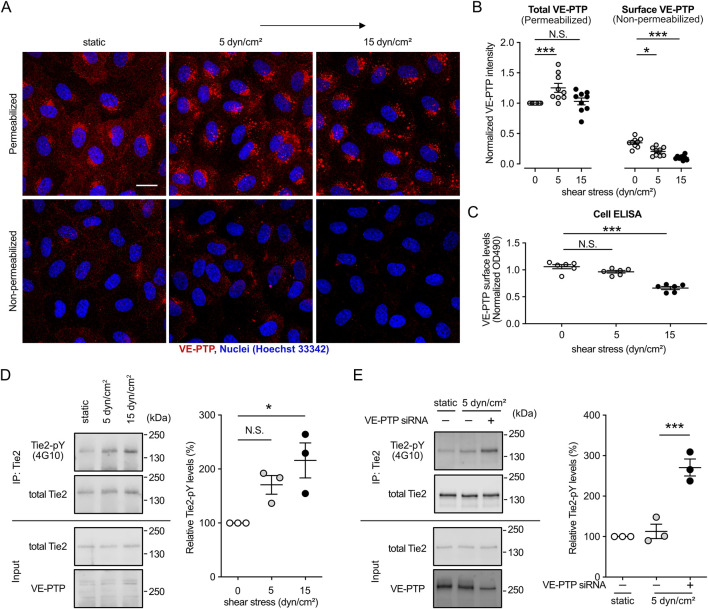
Shear stress magnitude determines the surface expression levels of VE-PTP, which negatively correlate with Tie-2 phosphorylation levels. **(A,B)** Effect of the magnitude of shear stress on the internalization of VE-PTP. HUVECs were exposed to shear stress at 5 dyn/cm^2^ or 15 dyn/cm^2^ for 30 min in EBM-2 flow medium, followed by staining for VE-PTP with or without permeabilization of TrtionX-100 **(A)**. Quantification of the signal intensity was conducted with ImageJ/Fiji **(B)**. **(C)** VE-PTP surface levels measured by cell-based ELISA assay after exposure of cells as in **(A)**. **(D)**. Effect of the magnitude of shear stress on the phosphorylation of Tie-2. Immunoprecipitants of Tie-2 (top) or cell lysate aliquots (bottom) were immunoblotted for the indicated antigens (indicated on the left). pY-Tie-2/total-Tie-2 level of static condition in each experiment is normalized to 1. **(E)** Effect of VE-PTP inhibition on the phosphorylation of Tie-2 at low shear stress. Immunoprecipitants of Tie-2 (top) or cell lysate aliquots (bottom) were immunoblotted for the indicated antigens (indicated on the left). pY-Tie-2/total-Tie-2 level of static condition in each experiment is normalized to 1. Mean ± SEM, n = 9 **(B)**, 6 **(C)**, or 3 **(D,E)** P values were calculated by two-way ANOVA followed by Dunnett’s multiple tests **(B–D)** or by two-tailed Weltch’s t-test **(E)**. Scale bars: 20 μm **(A)**.

We have recently demonstrated that shear stress induces endocytosis of VE-PTP leading to reduced levels of VE-PTP on the cell surface, which results in increased phosphorylation of Tie-2 (Shirakura et al., 2023). In addition, we have reported that Tie-2 signaling is essential for cell alignment and elongation (Ghanbarpour Houshangi et al., 2025). Based on these studies, we now asked whether sensing of increasing magnitudes of shear stress levels would lead to inversely proportional levels of VE-PTP on the cell surface. To examine this, we exposed HUVECs to 5 and 15 dyn/cm^2^ of shear stress for 30 min, followed by treatment with or without TritonX-100 and antibody staining for VE-PTP. Staining of detergent permeabilized cells allowed to detect the accumulation of VE-PTP in intracellular vesicles of cells exposed to 5 and 15 dyn/cm^2^ (Figures 2A,B). Staining of non-permeabilized cells revealed that VE-PTP surface levels decreased gradually, with increasing levels of shear stress (Figures 2A,B). In agreement with these results, a cell surface ELISA assay showed that an increase of shear stress from 5 to 15 dyn/cm^2^ led to a decrease of VE-PTP levels on the cell surface (Figure 2C). Such a negative correlation of VE-PTP surface levels and fluid shear stress levels was still observed after exposure of HUVEC to shear stress of different magnitude for 24 h (Supplementary Figures S2A,B). Collectively, these results suggest that fluid shear stress levels determine VE-PTP surface levels in an inversely proportional and magnitude-dependent manner.

We next investigated whether VE-PTP surface levels determine Tie-2 phosphorylation levels. To this end, we exposed HUVEC to static culture conditions or 5 and 15 dyn/cm^2^ fluid shear stress followed by immunoblotting Tie-2 immunoprecipitants with the pan phospho-tyrosine antibody 4G10. As shown in Figure 2D, tyrosine phosphorylation of Tie-2 increased gradually with increased shear stress. The increase at 15 dyn/cm^2^ was significant, while the increase at 5 dyn/cm^2^ just missed being significant. We further investigated whether the less phosphorylation of Tie-2 at 5 dyn/cm^2^ fluid shear stress is due to the less VE-PTP internalization. We exposed HUVEC transfected with control or VE-PTP siRNA to 5 dyn/cm^2^ fluid shear stress and examined Tie-2 phosphorylation levels (Figure 2E). VE-PTP silencing significantly increased Tie-2 phosphorylation at 5 dyn/cm^2^ fluid shear stress.

We have recently found that the signaling mechanism whereby Tie-2 controls cell alignment is mediated by the stimulation of FOXO1 translocation from the nucleus into the cytosol which leads to activation of autophagy (Ghanbarpour Houshangi et al., 2025). Based on this, we now tested whether these effects would also gradually increase with a gradual increase of fluid shear stress. Comparing static conditions and three different shear stress levels (4, 15 and 59 dyn/cm^2^) we found that the levels of FOXO1 in the nucleus decreased. In addition, we detected in immunoblots that the levels of activated LC3B, a central protein in autophagy (Tanida et al., 2008; Li X. et al., 2020), increased gradually with increased shear stress (Supplementary Figures S2C,D). Together, these results establish that the magnitude of shear stress is a key parameter to adjust VE-PTP internalization and the downstream signaling effects on the Tie-2-FOXO1-autophagy axis.

### 3.3 Tie-2 is required for the role of VE-PTP in controlling endothelial cell morphological responses to fluid shear stress

**FIGURE 3:**
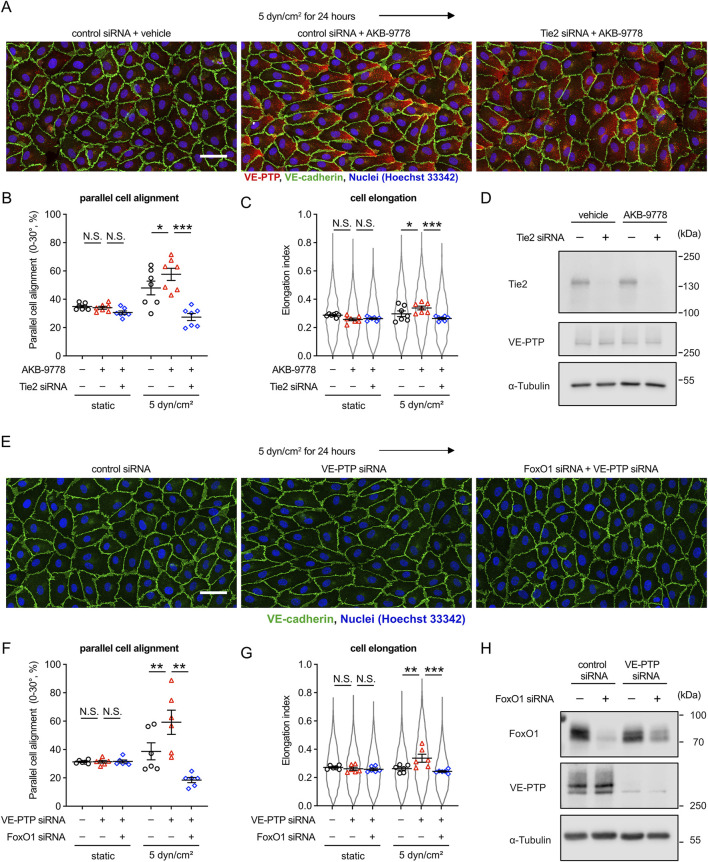
VE-PTP inhibition increases shear stress induced cellular morphological responses in a Tie-2-FOXO1 dependent way. **(A–C)** Effect of Tie-2 siRNA on AKB-9778-mediated promotion of cell alignment and elongation in 5 dyn/cm^2^ of shear stress. HUVECs transfected with control or Tie-2 siRNA were exposed to shear stress for 24 h with or without AKB-9778 in EBM-2 flow medium. The resulting cells were stained for VE-cadherin (green) and VE-PTP (red) **(A)**. Percentage of parallel cell alignment **(B)** and elongation **(C)** were quantified with Fiji/ImageJ. **(D)** siRNA-mediated Tie-2 silencing efficiency. HUVECs transfected with control or Tie-2 siRNA were treated with AKB-9778 for 4 hours. The resulting cells were lysed and immunoblotted with antibodies against Tie-2, VE-PTP and α-Tubulin. **(E–G)** Effect of FoxO1 siRNA on VE-PTP siRNA-mediated promotion of cell alignment and elongation in 5 dyn/cm^2^ of shear stress. HUVECs transfected with control, VE-PTP or FoxO1 siRNA were exposed to shear stress for 24 h in EBM-2 flow medium. The resulting cells were stained for VE-cadherin (green), FoxO1 (red) and Hoechst (blue) **(E)**. Percentage of parallel cell alignment **(F)** and elongation **(G)** were quantified with Fiji/ImageJ. **(H)** siRNA-mediated FoxO1 and VE-PTP silencing efficiency. HUVECs were transfected with control, VE-PTP or FoxO1 siRNA. The resulting cells were lysed and immunoblotted with antibodies against FoxO1, VE-PTP and α-Tubulin. Mean ± SEM; n = 7 **(A–C)** or 4 **(E–G)**; P values are calculated with two-way ANOVA followed by Dunnett’s multiple tests (**(C)**; vs. control siRNA + AKB-9778 and **(F)**; vs. VE-PTP siRNA). Scale bars: 50 μm **(A,E)**.

Although we have shown that Tie-2 is required for cell alignment and elongation upon exposure to fluid shear stress (Ghanbarpour Houshangi et al., 2025) and we have shown here that VE-PTP is also needed (Figure 1), we did not yet know, whether this function of VE-PTP is indeed mediated by Tie-2. This is especially important since VEGFR2 and VE-cadherin are also well described substrates of VE-PTP. Therefore, we tested the effect of VE-PTP inhibitor AKB-9778 on the morphological response of HUVEC cells to 5 dyn/cm^2^ fluid shear stress after treating the cells with either Tie-2 or control siRNA. We found that AKB-9778 promoted the parallel cell alignment and elongation in HUVECs treated with control siRNA, whereas these effects were blocked for cells treated with Tie-2 siRNA (Figures 3A–C). We verified Tie-2 silencing efficiency by immunoblot (Figure 3D). In line with this, silencing FOXO1, a downstream target of Tie-2 signaling, blocked VE-PTP siRNA effects on cell alignment and elongation as well (Figures 3E–G). We verified FoxO1 and VE-PTP silencing efficiency by immunoblot (Figure 3H) Thus, VE-PTP regulates the endothelial cell morphological responses to fluid shear stress through Tie-2 activity.

Since we had found that VE-PTP silencing did not affect cell alignment and elongation perpendicular to the direction of flow when cells we exposed to 30 dyn/cm^2^, we tested whether Tie-2 is relevant. Interestingly, we found that silencing of Tie-2 by siRNA did block these morphological cellular responses (Supplementary Figure S3). This result shows that Tie-2 is relevant for this cellular morphological response to high shear stress, but the effect cannot be further increased by interference with VE-PTP activity.

### 3.4 Tie-2 activation driven by Angpt1 alters the endothelial morphological responses similarly as VE-PTP inhibition

**FIGURE 4:**
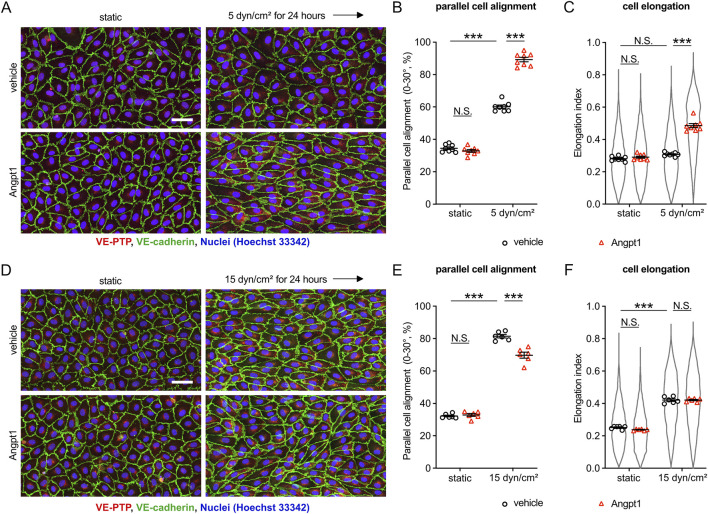
Tie-2 activation induced by Angpt1 alters cellular morphological adaptation similarly to VE-PTP inhibition. **(A–F)** Effect of Angpt1 on cell alignment and cell elongation under 5 dyn/cm^2^ **(A–C)** or 15 dyn/cm2 **(D–F)** of shear stress. HUVECs were exposed to indicated shear stress for 24 h with or without Angpt1 at 600 ng/mL in EBM-2 flow medium. The resulting cells were stained for VE-cadherin (green) and VE-PTP (red) **(A,D)**. Cell alignment **(B,E)** and elongation **(C,F)** were quantified with Fiji/ImageJ. Mean ± SEM n = 8 **(A–C)** or 6 **(D–F)**. P values are calculated with two-way ANOVA followed by Tukey’s multiple tests **(C,F)**. Scale bars: 50 μm **(A,D)**.

Our results so far suggested that VE-PTP inhibition increases the sensitivity of endothelial cells to fluid shear stress by increasing the activation level of Tie-2. To verify this conclusion, we tested whether the Tie-2 agonist Angpt1 would have similar effects as inhibition of VE-PTP. To this end, we exposed HUVEC to 5 dyn/cm^2^ fluid shear stress in the presence of Angpt1 or vehicle alone. We found that Angpt1 significantly promoted parallel cell alignment and elongation (Figures 4A–C). Repeating similar experiments under higher fluid shear stress (15 dyn/cm^2^) resulted in an inhibitory effect of Angpt1 on parallel cell alignment and no effect on cell elongation (Figures 4D–F), similarly as we found for VE-PTP siRNA (Figures 1D–F). Taken together, these results further verify that the Tie-2 activation level determines the sensitivity to shear stress levels, and VE-PTP acts via Tie-2 in this process.

### 3.5 VE-PTP-Tie-2 regulate cell morphological responses independent of the mechanosensory complex consisting of VE-cadherin, PECAM1 and VEGFR2

**FIGURE 5:**
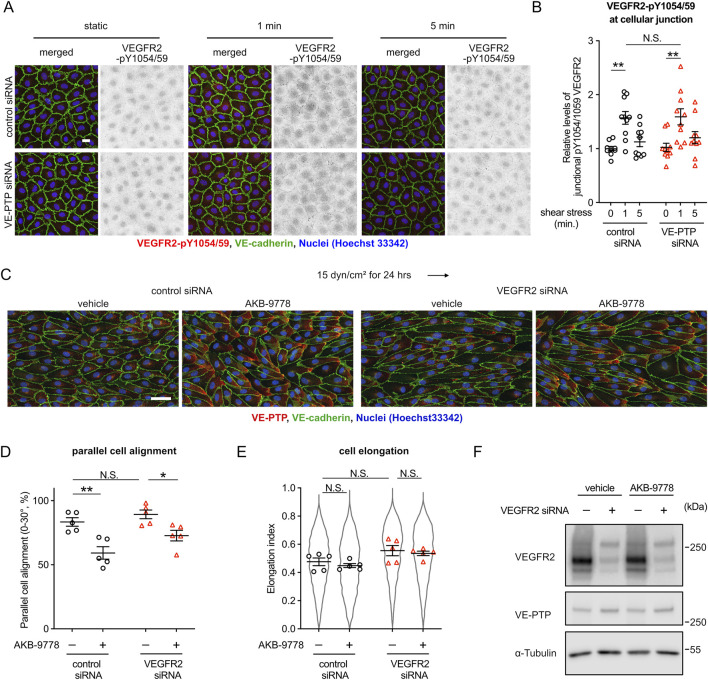
VE-PTP-Tie-2 signaling controls cell morphology independent of VEGFR2. **(A,B)** VE-PTP silencing does not affect the activation of VEGFR2 by shear stress. HUVECs treated with control or VE-PTP siRNA were exposed to shear stress at 15 dyn/cm^2^ for 1 or 5 min in EBM-2 flow medium, followed by staining for VEGFR2-pY1054/59 (red) and VE-cadherin (green) **(A)**. The signal intensity of VEGFR2-pY1054/59 at cellular junctions was quantified with ImageJ/Fiji **(B)**. Mean ± SEM n = 10. P values are calculated by two-way ANOVA followed by Tukey’s multiple tests. **(C–E)** VEGFR2 silencing does not affect AKB-9778-mediated impairment of cell alignment at 15 dyn/cm^2^ of shear stress. HUVECs transfected with control or VEGFR2 siRNA were exposed to shear stress for 24 h with or without AKB-9778 in EBM-2 flow medium. The resulting cells were stained for VE-cadherin (green) and VE-PTP (red) **(C)**. Percentage of parallel cell alignment **(D)** and elongation **(E)** were quantified with Fiji/ImageJ. **(F)** siRNA-mediated VEGFR2 silencing efficiency. HUVECs transfected with control or VEGFR2 siRNA were treated with AKB-9778 for 4 hours. The resulting cells were lysed and immunoblotted with antibodies against VEGFR2, VE-PTP and α-Tubulin. Mean ± SEM n = 5. P values are calculated with two-way ANOVA followed by Tukey’s multiple tests **(D,E)**. Scale bars: 20 μm **(A)** and 50 μm **(C)**.

It is well known that the mechanosensory complex (PECAM-1, VE-cadherin, VEGFR2/3) controls the sensitivity to shear stress (Baeyens et al., 2015). Therefore, we asked how the VE-PTP-Tie-2 mediated control of shear stress sensitivity is related to the function of the mechanosensitive complex. First, we tested whether VE-PTP silencing would affect shear stress induced phosphorylation of VEGFR2. Exposing HUVEC for 1 min or for 5 min to 15 dyn/cm^2^ fluid shear stress, we found that an antibody against pY1054/59 of VEGFR2 allowed specific staining at junctions after 1 min of flow (Figures 5A,B), which is in agreement with earlier findings (Coon et al., 2015). Silencing of VE-PTP by siRNA treatment did not affect this staining. (Figures 5A,B). We confirmed the specificity of the antibody by staining HUVECs after incubation under static conditions for 10 min with VEGF-A. Positive staining was seen with cells pretreated with control siRNA and was lost after pre-treatment with VEGFR2 siRNA (Supplementary Figures S4A,B). Likewise, staining of junctions with antibodies against VEGFR2-pY1054/59 after 1 min of fluid shear stress (15 dyn/cm^2^) was abrogated after pre-treatment with VEGFR2 siRNA (Supplementary Figures S4C,D).

Next, we analyzed the effects of VE-PTP inhibition to cell morphology in the absence of VEGFR2. Regardless of VEGFR2 expression, VE-PTP inhibition with AKB-9778 suppressed cell alignment and elongation induced by shear stress at 15 dyn/cm^2^ (Figures 5C–E). We verified VEGFR2 silencing efficiency by immunoblot (Figure 5F). This indicates that VE-PTP-Tie-2 signaling controls cell morphological response to fluid shear stress independently of the mechanosensory complex. Surprisingly, however, VEGFR2 silencing did not inhibit cell alignment and elongation in our experimental conditions. Rather VEGFR2 silencing slightly but not significantly enhanced cell alignment and elongation. Together, these results show that the VE-PTP/Tie-2 signaling axis acts independently of the mechanosensory complex in controlling the cellular morphological responses to fluid shear stress. In addition, the medium conditions we used here may have an effect on how VEGFR2 influences cell alignment to flow.

It was unexpected and surprising that VEGFR2 silencing did not inhibit cell alignment and that it even increased cell alignment in the presence of VEGF-A. In previous reports (Coon et al., 2015; Chen et al., 2024; Tanaka et al., 2024), the roles of the mechanosensory complex have been mainly studied with HUVEC in M199 medium, whereas we performed our flow experiments throughout our study in EBM-2 medium (Lonza). Thus, we hypothesized that the different media compositions might influence the role of VEGFR2 in flow induced cell alignment.

**FIGURE 6:**
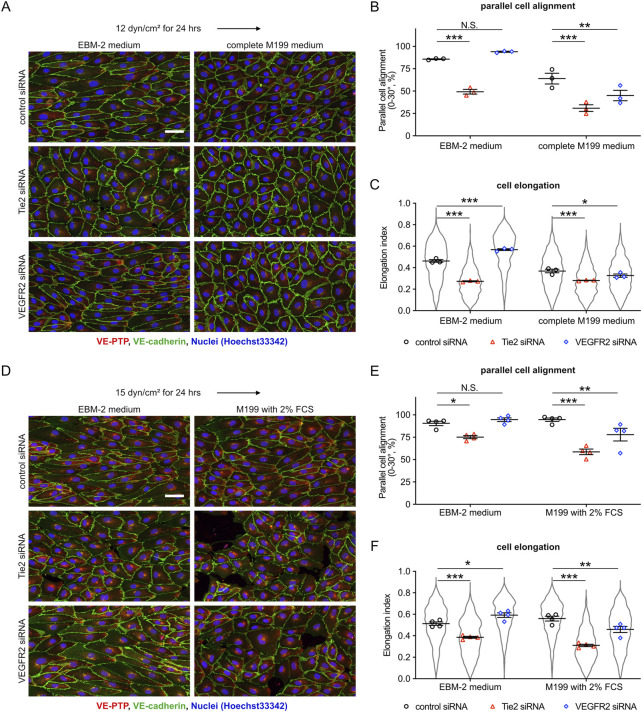
Cell culture media influence the role of VEGFR2, but not of Tie-2, in controlling shear stress-induced cellular morphological responses. **(A–C)** Comparing the effects of VEGFR2-and Tie-2-silencing on shear stress responses in different cell culture media. HUVEC transfected with either control, Tie-2 or VEGFR2 siRNA were exposed to shear stress at 12 dyn/cm^2^ in EBM-2 flow medium or complete M199 medium (containing 20% FBS, 50 μg/mL ECGS). The resulting cells were stained for VE-PTP (red) and VE-cadherin (green) **(A)**. Percentage of parallel cell alignment **(B)** and elongation **(C)** were quantified with ImageJ/Fiji. Mean ± SEM n = 3. P values are calculated with two-way ANOVA followed by Dunnett’s multiple tests (vs. control siRNA). **(D–F)** Similar as for **(A–C)**, except that here experiments were performed in M199 medium (only containing 2% FCS) and EBM-2 flow medium, and cells were exposed to 15 dyn/cm^2^ shear stress. Cells were stained as above **(D)**. Cell alignment **(E)** and elongation **(F)** were quantified with ImageJ/Fiji. Mean ± SEM n = 4. P values are calculated with two-way ANOVA followed by Dunnett’s multiple tests (vs. control siRNA). Scale bars: 50 μm **(A,D)**.

To test this, we compared the cellular responses in EBM-2 medium with those in complete M199 medium. HUVEC were exposed to 12 dyn/cm^2^ in each of the two media after pretreatment with either control, Tie-2 or VEGFR2 siRNA. We found that Tie-2 siRNA inhibited cell alignment and elongation regardless of the medium, whereas VEGFR2 siRNA suppressed cell alignment and elongation only in the complete M199 medium (Figures 6A–C). Complete M199 medium contains 20% FCS and several additional growth factors based on the addition of endothelial cell growth supplement (ECGS). In contrast, EBM-2 medium simply contained 2% FCS. To exclude differences in growth factors as a reason for the different cellular responses, we repeated our experiments with EBM-2 and M199 media each only containing 2% FCS without additional growth supplements added. We observed that a low percentage of HUVECs transfected with Tie-2 siRNA were detached in M199 media containing 2% FCS. Under these conditions, again, we found that Tie-2 siRNA suppressed cell alignment and elongation regardless of the medium, whereas VEGFR2 siRNA treatment suppressed cell alignment and elongation only in M199 medium, but not in EBM-2 medium (Figures 6D–F). These results indicate that the biochemical composition of the culture medium affects functioning of VEGFR2-based but not Tie-2-based sensing of fluid shear stress. Collectively, our results suggest that VE-PTP-Tie-2 is a novel pathway for the control of cell morphology, which responds in a quantitative way to different fluid shear stress magnitudes.

## 4 Discussion

Blood flow helps to control the diameter of blood vessels. Increased or decreased flow triggers outward or inward remodeling, respectively, to adjust lumen diameters and re-establish previous fluid shear stress conditions (Kamiya and Togawa, 1980; Kamiya et al., 1984; Langille and O’Donnell, 1986; Langille et al., 1989; Tronc et al., 1996; Tuttle et al., 2001). Since different types of blood vessels (arteries, capillaries, veins and lymphatic vessels) are exposed to different levels of fluid shear stress, this concept implies that endothelial cells of different blood vessel types differ in their sensitivity to fluid shear stress. In a pioneer study it was shown that the expression level of VEGFR3 – as part of the PECAM-1/VE-cadherin/VEGFR mechanosensory complex–determines the range of shear stress levels (or the flow set point) which can be sensed by certain types of endothelial cells and guides their cellular responses (Baeyens et al., 2015). The current study shows that VE-PTP/Tie-2 is another, new signaling system that determines sensitivity and cellular responsiveness to different shear stress magnitudes or flow set points. We found that VE-PTP cell surface expression and the activation of Tie-2 and downstream signaling effects are gradually affected in response to different shear stress magnitudes. In addition, the VE-PTP/Tie-2 signaling module controls changes in cell morphology in response to shear stress magnitude. Finally, we found that VE-PTP/Tie-2 determines shear stress sensitivity independent of VEGFR2 activity. Thus, we propose a novel mechanism consisting of VE-PTP and Tie-2 which determines the sensitivity of endothelial cells to shear stress magnitude.

The different flow conditions in different types of blood and lymphatic vessels require mechanisms that determine different flow set points in the endothelium of these vessels. VEGFR3 is more strongly expressed in lymphatic than in blood endothelium which can explain the higher sensitivity of lymphatic endothelial cells for low fluid shear stress levels (Baeyens et al., 2015). In addition, interfering *in vivo* with the expression level of VEGFR3 modulates aortic lumen diameter, consistent with flow dependent remodeling (Baeyens et al., 2015). Although not directly analyzed here, it is conceivable that differences in the signal intensity of VE-PTP/Tie-2 in different endothelial cells are also relevant for determining flow set points and influencing vascular remodeling *in vivo*. Fluid shear stress levels are tenfold higher in arteries than in veins (Papaioannou and Stefanadis, 2005). In line with this, VE-PTP levels are lower in veins than in arteries (Baumer et al., 2006) and expression levels of Tie-2 are higher in veins than in arteries (Chu et al., 2016). Thus, venular endothelium would be expected to be capable of stronger Tie-2 signaling levels than arterial endothelium, which could possibly be linked to higher sensitivity for low shear stress levels as they exist in veins. Furthermore, we showed previously that gene inactivation of VE-PTP *in vivo* in mice or interference with VE-PTP function *in vivo* leads to increased Tie-2 activation and vessel enlargement (Baumer et al., 2006; Dominguez et al., 2007; Winderlich et al., 2009). In addition, mutations rendering Tie-2 constitutively active are a cause of venous malformations, which are selectively detected in slow-flow vessels (Vikkula et al., 1996; Wouters et al., 2010; Natynki et al., 2015). Other studies showed that upregulation and activation of Tie-2 *in vivo* contribute to pathological vessel enlargement and vascular malformation (Zhou et al., 2021; Banerjee et al., 2023). These studies in combination with our findings reported here are in line with a concept that fluid shear stress influences VE-PTP-controlled Tie-2 activation levels which determine shear stress sensitivity and vascular remodeling.

Since VE-PTP dephosphorylates also other cell surface proteins and some of the best studied VE-PTP substrates, such as VEGFR2 and VE-cadherin, are even highly relevant for the control of fluid shear stress-induced cell alignment and elongation, it is important that we showed here that the role of VE-PTP as regulator of cell alignment is indeed exclusively regulated by Tie-2 and the Tie-2 downstream signaling target FOXO1. This is based on our finding that endothelial cells treated with the VE-PTP inhibitor AKB-9778 aligned better at low shear stress levels (5 dyn/cm^2^) and this effect was absolutely dependent on Tie-2. In line with this, the Tie-2 agonist Angpt1 had the same effect as the VE-PTP inhibitor. These results are in agreement with a previous study which reported that endothelial cells lacking SMAD4 align and elongate to low shear stress through upregulating Tie-2 expression levels (Banerjee et al., 2023).

**FIGURE 7:**
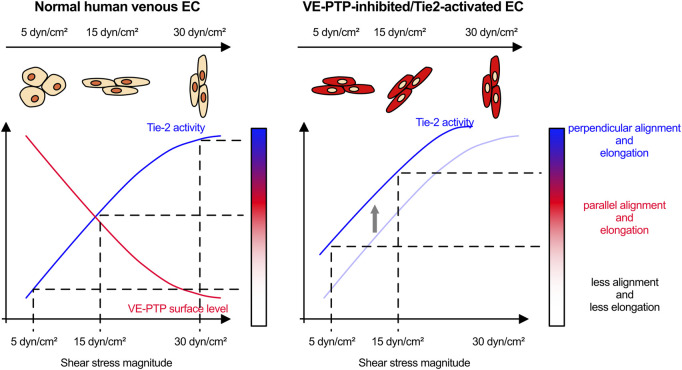
Proposed model how VE-PTP functions to determine the sensitivity to shear stress through Tie-2 activity.

At slightly higher physiological shear stress levels (15 dyn/cm^2^), inhibition of VE-PTP even impaired parallel cell alignment. While surprising at first glance, a potential explanation for this finding would be that VE-PTP inhibition leads at such shear stress levels to excessive Tie-2 activation which may create a condition at which already cells start to partially align in perpendicular orientation (see proposed mechanistic model Figure 7). Increasing shear stress levels to 30 dyn/cm^2^ induced cell alignment perpendicular to the direction of flow, which was an effect that required Tie-2, but not VE-PTP. It is conceivable that additional mechanisms exist which are independent of VE-PTP and could stimulate Tie-2 in a shear stress dependent way. An example for such a mechanism could be the Piezo1 driven influx of Ca^2+^ which was reported to trigger Anpt2 exocytosis which caused Tie1 shedding and thereby indirectly the activation of Tie-2 (Du et al., 2024). Another possible explanation could be that Tie-2 is required but not sufficient for the induction of perpendicular cell alignment. A previous study reported that E-selectin is involved in perpendicular cell alignment in lymphatic endothelial cells (Michalaki et al., 2020). E-selectin expression is upregulated by NFκB, which in turn is known to be activated by very low and high levels of shear stress (Baeyens et al., 2015). Although speculative, it is possible that such inflammatory molecules at the cell surface are acting in parallel with the VE-PTP/Tie-2 signaling mechanism to control cell alignment at higher magnitude of shear stress.

Given the fact that VEGFR2 can act as a substrate of VE-PTP in endothelial cells (Mellberg et al., 2009; Hayashi et al., 2013), it is intriguing that the role of VE-PTP as regulator of shear stress-controlled cell alignment was completely independent of VEGFR2, since silencing of VEGFR2 did not affect the AKB-9778 effects on cell morphological responses to shear stress. Surprisingly, in the medium we used for our study (EBM-2 medium), VEGFR2 silencing had no effect on shear stress sensitivity of HUVEC, although it became phosphorylated at Y1054/59 upon short term exposure to fluid shear stress. In contrast, comparing the relevance of VEGFR2 and Tie-2 for cell alignment in another medium (M199) revealed that each tyrosine kinase receptor was involved in cell alignment. It is presently unknown why VEGFR2 was only relevant in one of the two media whereas Tie-2 controlled cell alignment in the direction of flow in both media. Growth factor content could be excluded as a reason. Since shear stress did stimulate the phosphorylation of VEGFR2 in EBM-2 medium, it may be rather downstream effectors of this receptor which function differently in this medium. VEGFR2 signaling is well studied and there would be several potential candidates for future investigations (Simons et al., 2016). Whatever the reason is for the exclusive function of Tie-2 in EBM-2 medium, our results clearly show that the VE-PTP/Tie-2 system can act independently of the VEGFR2-containing mechanosensory complex and thus represents a novel additional mechanism for the control of cell alignment.

Collectively, our study shows that VE-PTP/Tie-2 represents a novel signaling mechanism by which endothelial cells distinguish and differentially respond to different shear stress levels (Figure 7). It is intriguing to speculate that this may be relevant for the roles of VE-PTP and Tie-2 for determining vessel diameter during development (Baumer et al., 2006; Dominguez et al., 2007; Winderlich et al., 2009) and the role of Tie-2 in venous malformations (Vikkula et al., 1996; Wouters et al., 2010; Natynki et al., 2015), a concept which will be interesting to study in more detail in the future.

## Data Availability

The raw data supporting the conclusions of this article will be made available by the authors, without undue reservation.
