# Supplementary figures and images for: VE-PTP controls a fluid shear stress set point that governs cell morphological responses through Tie-2
Source: Front Cell Dev Biol. 2025 Jul 4;13:1603517. doi: 10.3389/fcell.2025.1603517 (PMC12271748; doi:10.3389/fcell.2025.1603517)

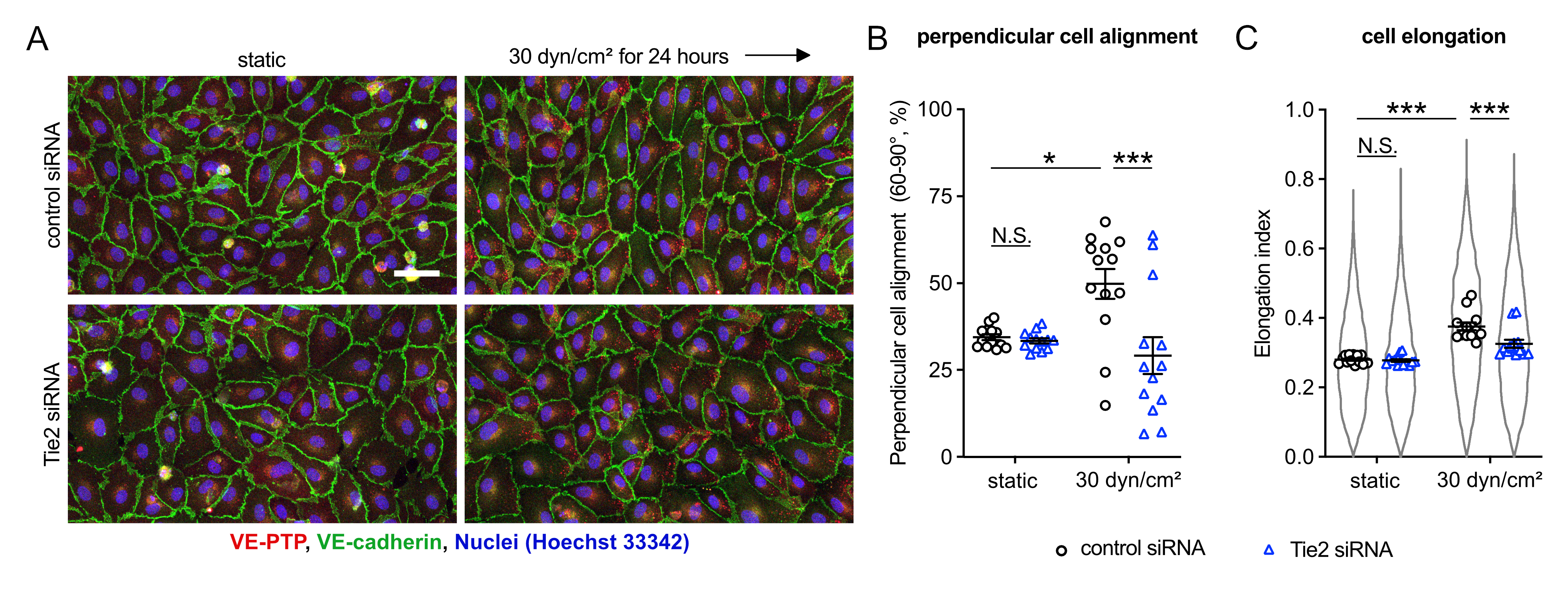

Supplement: Supplementary file 1 [file Image3.tiff]

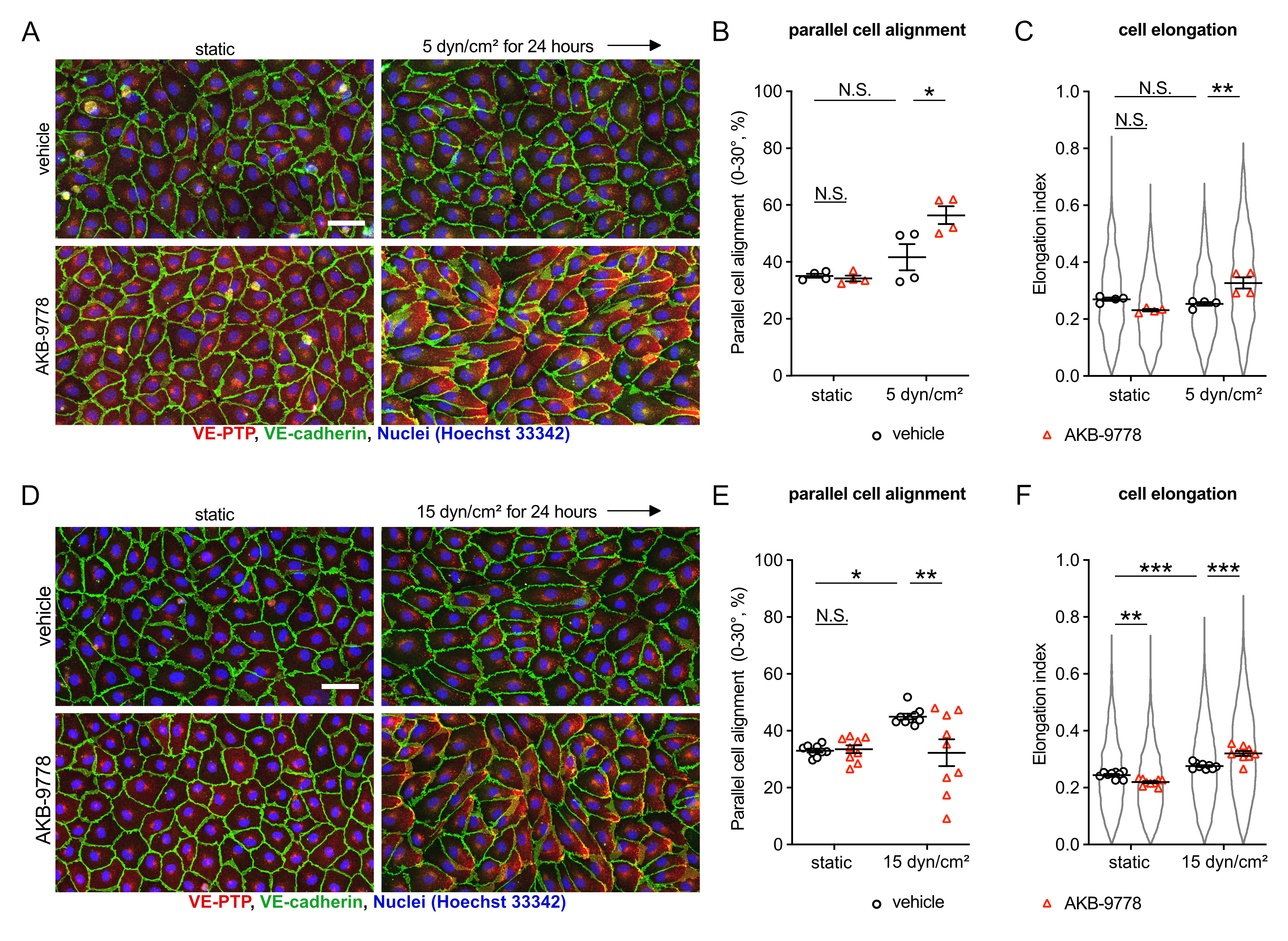

Supplement: Supplementary file 2 [file Image1.tiff]

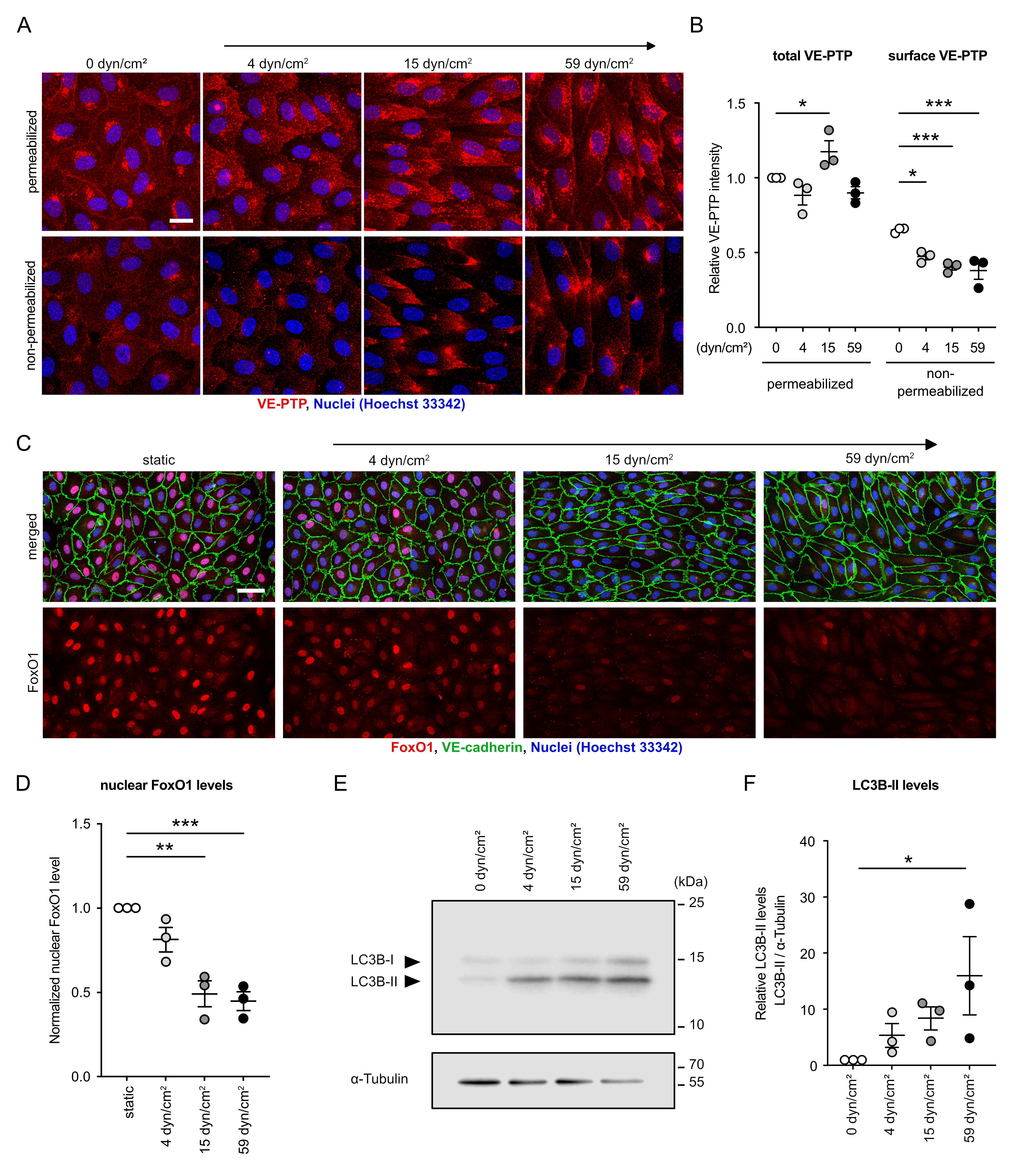

Supplement: Supplementary file 3 [file Image2.tiff]

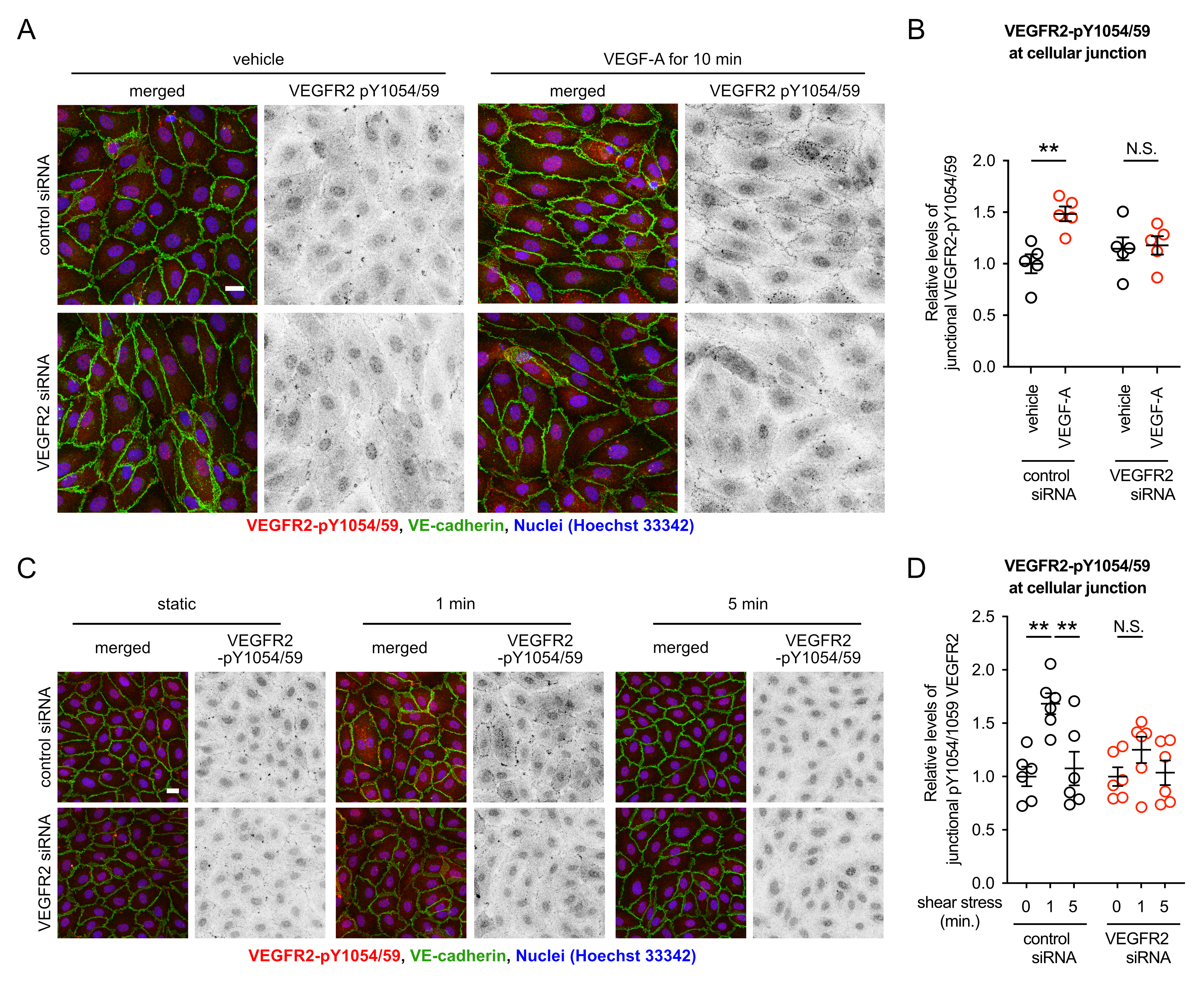

Supplement: Supplementary file 4 [file Image4.tiff]
